# Lovastatin, not Simvastatin, Corrects Core Phenotypes in the Fragile X Mouse Model

**DOI:** 10.1523/ENEURO.0097-19.2019

**Published:** 2019-06-10

**Authors:** Melania Muscas, Susana R. Louros, Emily K. Osterweil

**Affiliations:** 1 Centre for Discovery Brain Sciences, Simons Initiative for the Developing Brain, University of Edinburgh, Edinburgh EH8 9XD, United Kingdom

**Keywords:** ERK, FMR1, fragile X, lovastatin

## Abstract

The cholesterol-lowering drug lovastatin corrects neurological phenotypes in animal models of fragile X syndrome (FX), a commonly identified genetic cause of autism and intellectual disability (ID). The therapeutic efficacy of lovastatin is being tested in clinical trials for FX; however, the structurally similar drug simvastatin has been proposed as an alternative due to an increased potency and brain penetrance. Here, we perform a side-by-side comparison of the effects of lovastatin and simvastatin treatment on two core phenotypes in *Fmr1^-/y^* mice versus WT littermates: excessive hippocampal protein synthesis and susceptibility to audiogenic seizures (AGSs). We find that simvastatin does not correct excessive hippocampal protein synthesis in the *Fmr1^-/y^* hippocampus at any dose tested. In fact, simvastatin significantly increases protein synthesis in both *Fmr1^-/y^* and WT. Moreover, injection of simvastatin does not reduce AGS in the *Fmr1^-/y^* mouse, while lovastatin significantly reduces AGS incidence and severity versus vehicle-treated animals. These results show that unlike lovastatin, simvastatin does not correct core phenotypes in the *Fmr1^-/y^* mouse model.

## Visual Abstract

**Figure:**
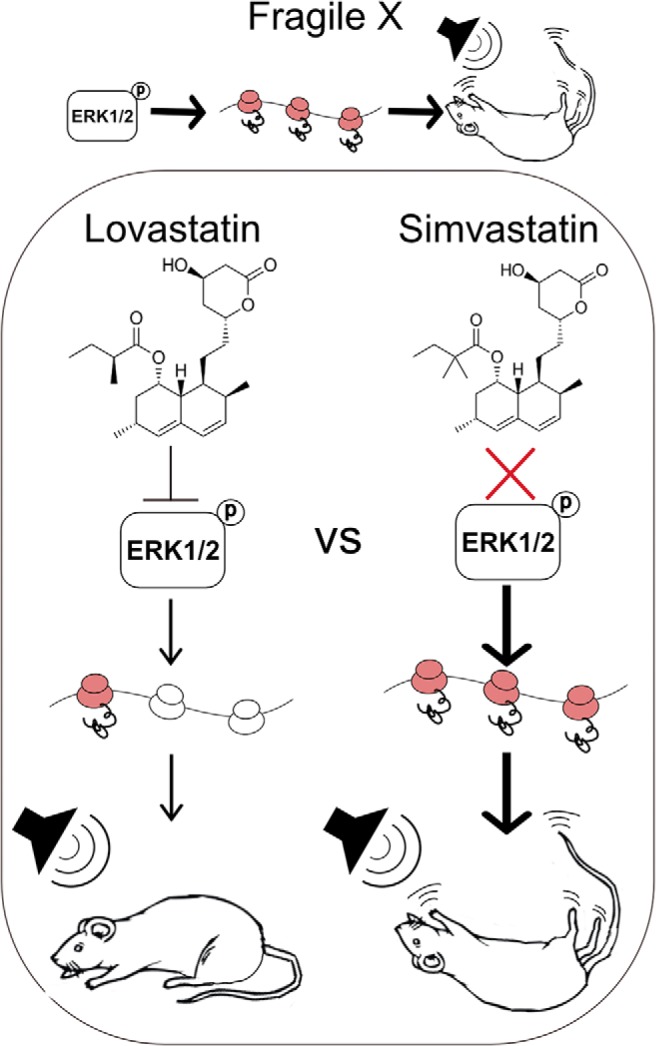

## Significance Statement

The statin drug lovastatin is in clinical trials for the treatment of fragile X syndrome (FX), and the structurally similar drug simvastatin has been proposed as a viable alternative. This study compares the efficacy of these drugs for ameliorating two major phenotypes in the FX mouse model and shows that although lovastatin is effective in correcting excessive protein synthesis and audiogenic seizures (AGSs), simvastatin fails to correct either phenotype. These results suggest caution should be used when assuming simvastatin is a suitable substitute for lovastatin with respect to the treatment of FX or other neurodevelopmental disorders.

## Introduction

Fragile X syndrome (FX) is a monogenic neurodevelopmental disorder characterized by severe intellectual disability (ID), autism, hypersensitivity to sensory stimulation and epilepsy (Lozano et al., 2014). FX occurs in 1:4000 males and 1:8000 females, making it one of the most commonly identified genetic causes of autism and ID (Hagerman et al., 2009; Lozano et al., 2014). The *FMR1* gene mutated in FX encodes fragile X mental retardation protein (FMRP), which represses mRNA translation in neurons (Ashley et al., 1993; Darnell et al., 2011). Studies of the *Fmr1^-/y^* mouse model of FX reveal that excessive cerebral protein synthesis is a major consequence of *Fmr1* deletion (Qin et al., 2005; Dölen et al., 2007; Berry-Kravis et al., 2017; Stoppel et al., 2017b), which can be normalized through antagonism of metabotropic glutamate receptor 5 (mGlu_5_) or the downstream extracellular regulated kinase 1/2 (ERK1/2) MAP kinase and mammalian target of rapamycin (mTOR)-p70 S6 kinase (p70S6K) signaling pathways (Dölen et al., 2007; Osterweil et al., 2010; Sharma et al., 2010; Michalon et al., 2012; Wang et al., 2012). These strategies correct multiple neurologic phenotypes in the *Fmr1^-/y^* mouse, including an enhanced susceptibility to audiogenic seizures (AGSs; Bear et al., 2004; Dölen et al., 2007; Osterweil et al., 2010; Stoppel et al., 2017a). The current challenge is to successfully transition these therapeutic approaches to the clinic.

Previous work shows that the statin drug lovastatin, currently used for the treatment of high cholesterol in adults and children, resolves neuropathology in the *Fmr1^-/y^* mouse model (Osterweil et al., 2013). Lovastatin normalizes protein synthesis by reducing the farnesylation and subsequent activation of the GTPase Ras, which lies upstream of the ERK1/2 signaling pathway (Schafer et al., 1989; Mendola and Backer, 1990). By this mechanism, lovastatin has also been shown to successfully correct electrophysiological and behavioral phenotypes in the mouse model of neurofibromatosis type 1 (NF1), a neurodevelopmental disorder of excess Ras (Li et al., 2005). In contrast to ERK1/2, the mTOR-p70S6K pathway activated by the GTPase Rheb is not altered by lovastatin suggesting the impact on farnesylation does not extend to all targets (Osterweil et al., 2013).

In the *Fmr1^-/y^* mouse, the reduction of Ras-ERK1/2 by lovastatin ameliorates hippocampal epileptogenesis and neocortical hyperexcitability and significantly reduces the incidence of AGS (Osterweil et al., 2013). The AGS phenotype is one of the most robust behavioral phenotypes seen in the *Fmr1^-/y^* mouse, and it models the epilepsy observed in FX patients (Musumeci et al., 2000; Berry-Kravis, 2002). Several previous studies have used AGS as a benchmark for determining the efficacy of potential treatment strategies, consistently finding a positive correlation between treatment efficacy at reducing seizure incidence and correction of other pathologies (Yan et al., 2005; Dölen et al., 2007; Osterweil et al., 2010, 2013; Busquets-Garcia et al., 2013; King and Jope, 2013). Based on the positive outcome with lovastatin in *Fmr1^-/y^* animal models, two open-label clinical trials tested the viability of lovastatin for the treatment of FX (Çaku et al., 2014; Pellerin et al., 2016). Both studies revealed a significant improvement with lovastatin treatment, and a double-blind placebo-controlled trial is ongoing (Berry-Kravis et al., 2017).

Interestingly, the availability of lovastatin is not widespread in Europe and is not licensed for use in the United Kingdom. Instead, the drug simvastatin has been proposed as an alternative therapeutic. Simvastatin is a structurally similar derivative of lovastatin that is twice as potent, with a daily dose of only 10 mg reducing cholesterol by 25–30% compared to 20 mg of lovastatin (Jones et al., 1998; Schaefer et al., 2004; Neuvonen et al., 2008). Simvastatin is also more brain penetrant than lovastatin, suggesting it may be a better option for neurologic indications (Tsuji et al., 1993). However, simvastatin has not been investigated in the *Fmr1^-/y^* model, and the impact on Ras-ERK1/2 signaling in the brain is not well established. This information is critical, as clinical trials in NF1 have recently shown that lovastatin has a beneficial impact on cognitive function whereas simvastatin does not (Krab et al., 2008; Alabama-Birmingham and NCI, 2009, 2010; van der Vaart et al., 2013; Bearden et al., 2016; Payne et al., 2016).

In this study, we performed a side-by-side comparison of lovastatin and simvastatin to answer the simple but important question of whether there is a similar rescue of pathology in the *Fmr1^-/y^* mouse. We focused on two core phenotypes in the *Fmr1^-/y^* model: excessive protein synthesis and enhanced susceptibility to AGS. Importantly, our results clearly show that lovastatin, but not simvastatin, is effective in reducing ERK1/2 activity and normalizing protein synthesis in the *Fmr1^-/y^* hippocampus. This suggests that simvastatin acts via a different mechanism from lovastatin with respect to ERK1/2-driven protein synthesis in the brain. To examine whether there was a similar impact on pathology, we performed a thorough AGS analysis using multiple doses of simvastatin. The results of these experiments show that simvastatin does not reduce the incidence or severity of AGS in the *Fmr1^-/y^* mouse under conditions where lovastatin is significantly effective. Together, this evidence suggests simvastatin may not be a suitable replacement for lovastatin with respect to the treatment of FX.

## Materials and Methods

### Mice

All mice tested were male and were naive to drug and behavioral testing before experimentation. Mice were group housed with unrestricted food and water access and a 12/12 h light/dark cycle. Room temperature was maintained at 21 ± 2°C. All animal procedures were performed in accordance with the University of Edinburgh animal care committee’s regulations and the United Kingdom Animals Act. *Fmr1^-/y^* mice (The Jackson Laboratory 003025, RRID:IMSR_JAX:003025) were maintained on either a C57BL/6J (Charles River) or a mixed C57BL/6J x FVB background (C57BL/6J backcrossed to FVB by two generations).

### Metabolic labeling

Hippocampal slices were prepared from male littermate wild-type (WT) and *Fmr1^-/y^* (KO) C57BL/6J mice [postnatal day (P)25–P32], in an interleaved fashion, with the experimenter blind to genotype. Mice were anaesthetized with isoflurane, and the hippocampus was rapidly dissected in ice-cold ACSF (124 mM NaCl, 3 mM KCl, 1.25 mM NaH_2_PO_4_, 26 mM NaHCO_3_, 10 mM dextrose, 1 mM MgCl_2_, and 2 mM CaCl_2_, saturated with 95% O_2_ and 5% CO_2_). Slices (500 µm thick) were prepared using a Stoelting Tissue Slicer and transferred into 32.5°C ACSF (saturated with 95% O_2_ and 5% CO_2_) within 5 min. Slices were incubated in 32.5°C ACSF for 4 h to allow for recovery of protein synthesis then transferred to ACSF containing 25 μM Actinomycin D (Tocris) plus either vehicle (0.05% DMSO in ddH_2_O), 50 μM lovastatin active form (CAS 75225-50-2; Calbiochem Merck Millipore), or 0.1–5 μM simvastatin active form (CAS 101314-97-0; Cayman Chemical) for 30 min. To measure new protein synthesis, slices were then transferred to fresh ACSF with 10 µCi/ml ^35^S-Met/Cys (PerkinElmer) containing vehicle (veh) or drug for another 30 min.

After labeling, slices were homogenized in ice-cold buffer (10 mM HEPES, pH 7.4, 2 mM EDTA, 2 mM EGTA, 1% Triton X-100, protease inhibitors, and phosphatase inhibitors) and incubated in trichloroacetic acid (TCA; 10% final) for 10 min on ice before being centrifuged at 16,000 rpm for 10 min. The pellet was washed in ice-cold ddH_2_O and resuspended in 1 N NaOH until dissolved, and the pH was readjusted to neutral using 0.33 N HCl. Triplicates of each sample were subjected to scintillation counting and protein concentration assay kit (Bio-Rad). Counts per minute (CPM) were divided by protein concentration, and this was normalized to the CPM from the ACSF used for incubation. For display purposes, example slice homogenates were resolved on SDS-PAGE gels, transferred to nitrocellulose and exposed to a phosphorimaging screen (GE Healthcare). Phosphorimages were acquired using a Typhoon scanner (GE Healthcare) and compared to total protein staining of the same membrane.

### Immunoblotting

Samples were loaded on SDS-PAGE gels, with all conditions per littermate pair (i.e., WT veh, KO veh, WT drug, KO drug) present on the same gel (Extended Data Fig. 2-1). Samples were coded such that the experimenter was blinded to genotype and treatment. Gels were transferred to nitrocellulose and stained for total protein with the Memcode Reversible staining kit (Pierce). To immunoblot for ERK1/2 and p70S6K in the same samples, membranes were cut at 75, 50, and 37 kDa as shown in Extended Data Figure 2-1. For membranes probed for p-p70S6K, the portion of membrane above 75 kDa was removed to eliminate the background p85S6K band recognized by this antibody. Each membrane was then blo*c*ked with 5% BSA in TBS + 0.1% Tween 20 and incubated in primary antibody overnight at 4°C [Cell Signaling Technology; phospho-ERK1/2 (Thr202/Tyr204) 1:2000 (#9106, RRID:AB_331768), ERK1/2 1:2000 (#9102, RRID:AB_330744), phospho-p70S6K (Thr389) 1:1000 (#9234, RRID:AB_2269803), p70S6K 1:1000 (#2708, RRID:AB_390722); Extended Data Fig. 2-1]. Membranes were then washed, incubated with HRP-conjugated secondary antibodies for 30 min (Cell Signaling; RRID:AB_330924 and RRID:AB_2099233), and developed with Clarity ECL (Bio-Rad). Densitometry was performed on scanned blot films using Image Studio Lite software, RRID: SCR_013715.

**Figure 1.**
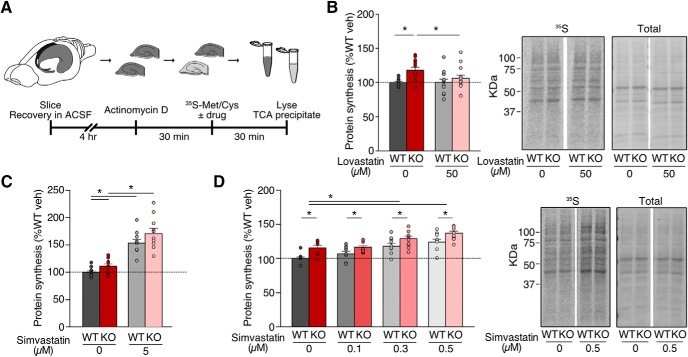
Simvastatin exaggerates excessive protein synthesis in the *Fmr1^-/y^* hippocampus. Slices were prepared from WT and *Fmr1^-/y^* hippocampi and incubated in vehicle, lovastatin, or simvastatin at different concentrations. ***A***, Schematic shows time course for metabolic labeling experiments of hippocampal slices. ***B***, Lovastatin significantly decreases protein synthesis in *Fmr1^-/y^* slices to WT levels (ANOVA genotype *p = 0.0106; Sidak’s WT veh vs KO veh *p = 0.0032, KO veh vs KO lova *p = 0.0368; *n* = 12). ***C***, Simvastatin raises protein synthesis in both WT and *Fmr1^-/y^* slices at 5 μM (ANOVA treatment *p < 0.0001, genotype *p = 0.0294; Sidak’s WT veh vs 5 μM *p = 0.0001, KO veh vs 5 μM *p < 0.0001; *n* = 10). ***D***, Simvastatin raises protein synthesis at 0.1–0.5 μM, exaggerating the excessive protein synthesis phenotype (ANOVA treatment *p < 0.0001, genotype *p = 0.0068; Sidak’s WT veh vs 0.3 μM *p = 0.0002, WT veh vs 0.5 μM *p < 0.0001, KO veh vs 0.3 μM *p = 0.0035, KO veh vs 0.5 μM *p < 0.0001, WT veh vs KO veh *p = 0.0005, WT 0.1 μM vs KO 0.1 μM *p = 0.0406, WT 0.3 μM vs KO 0.3 μM *p = 0.0115, WT 0.5 μM vs KO 0.5 μM *p = 0.0038; *n* = 9). Representative samples were run on SDS-PAGE gels and transferred to membranes. Example phosphorimages of ^35^S-labeled proteins and total protein staining of the same membrane are shown. Error bars = SEM. *N* = littermate pairs. *Figure Contributions*: Melania Muscas and Susana R. Louros performed the experiments and analyzed the data.

**Figure 2.**
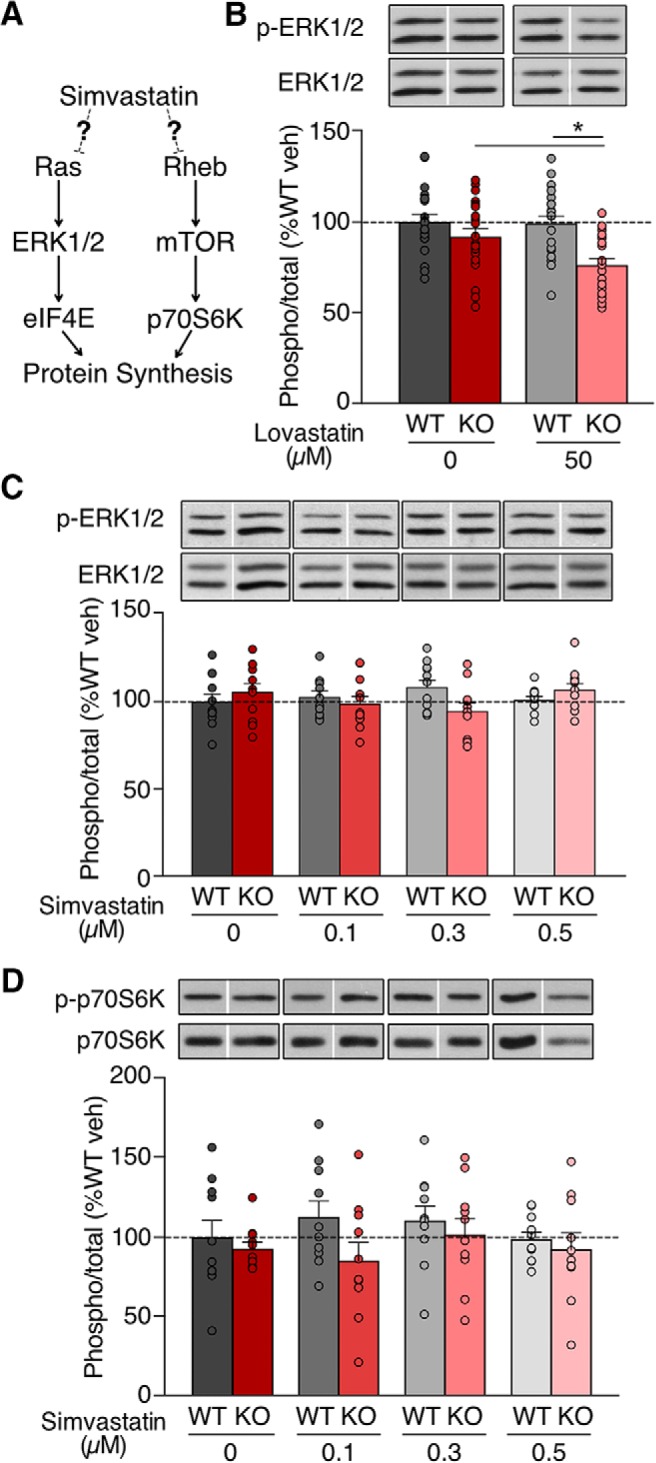
Simvastatin does not reduce ERK1/2 or mTORC1 activation in the *Fmr1^-/y^* hippocampus. ***A***, Diagram shows the potential impact of simvastatin on Ras-ERK1/2 and Rheb-mTOR-signaling pathways. ***B***, *Fmr1^-/y^* slices incubated with 50 µM lovastatin show a significant reduction in ERK1/2 phosphorylation (ANOVA genotype *p = 0.0146; Sidak’s KO veh vs KO lova *p = 0.0048; *n* = 19). ***C***, Simvastatin treatment does not reduce ERK1/2 phosphorylation in *Fmr1^-/y^* or WT slices (ANOVA treatment *p* = 0.8761, genotype *p* = 0.7010; *n* = 11). ***D***, Simvastatin treatment does not reduce phosphorylation of p70S6K in WT or *Fmr1^-/y^* slices (ANOVA treatment *p* = 0.6206, genotype *p* = 0.2860; *n* = 10). Representative bands were cropped from original blots as indicated by blank spaces. Original blots are shown in Extended Data Figure 2-1. Error bars = SEM. *N* = littermate pairs. *Figure Contributions*: Melania Muscas performed the experiments and analyzed the data.

10.1523/ENEURO.0097-19.2019.f2-1 Extended Data Figure 2-1 ***A***, Original immunoblots used for representative images in Figure 2. ***B***, Memcode-stained membranes were cut at 75 , 50, and 37 kDa to allow for analysis of ERK1/2 and p70S6K activation in the same samples. This strategy also removed p85S6K to prevent background binding of the p-p70S6K antibody. ***C***, Membranes used for analysis of p70S6K and ERK1/2 activation are shown. *Figure Contributions*: Melania Muscas and Susana R. Louros performed the experiments and analyzed the data. Download Figure 2-1, TIF file.

To compare phopho to total for each target in the same lane, membranes developed for phospho [i.e., phosphorylated (p-)ERK1/2] were stripped and reprobed for total (i.e., ERK1/2). Phosphorylation of target proteins was calculated as a ratio of phospho to total. To correct for blot-to-blot variance, each signal was normalized to the average signal of all lanes on the same blot. Values are shown as a percentage of average WT vehicle for graphical purposes. All membranes were analyzed with experimenter blind to genotype and treatment.

### AGSs

Test cohorts were counterbalanced for genotype and treatment. Naive WT and *Fmr1^-/y^* male P18–P29 mice bred on a mixed C57BL/6J x FVB background were weighed and injected intraperitoneally with 3 mg/kg simvastatin prodrug (CAS 79902-63-9), 50 mg/kg simvastatin active form (CAS 101314-97-0), or 100 mg/kg lovastatin active form (CAS 75225-50-2) or respective vehicle (3%, 20%, or 50% DMSO + 10% Tween 80 in PBS). Animals were then transferred to a quiet (<60-dB ambient sound) room for 1 h. For testing, animals were moved to a transparent test chamber equipped with speakers and a webcam and allowed to habituate for 1 min. Audiogenic stimulation (recorded sampling of a modified personal alarm) was passed through an amplifier and 2 × 50-W speakers (KRK Rokit RP5 G3 Active Studio Monitor) to produce a stimulus of >130 dB for 2 min. A decibel meter was placed at a standard distance from the speakers to ensure a stable emission of sound throughout each session. Incidence and severity of seizures was scored and video files for each session were saved. Latency was measured as the number of seconds between onset of the AGS stimulus and appearance of the first seizure. Stages of AGS severity were assigned according to previous work as follows: (1) wild running (WR; pronounced, undirected running and thrashing), (2) clonic seizure (violent spasms accompanied by loss of balance), or (3) tonic seizure (loss of movement and postural rigidity in limbs and tail). Any animal that reached tonic seizure was immediately humanely killed. All injections, testing and scoring was performed with the experimenter blind to genotype and treatment.

### Statistics

Statistical testing was performed using GraphPad Prism 6 software, RRID: SCR_002798. For biochemistry experiments, outliers >2 SD from the mean were removed and significance determined by repeated measures two-way ANOVA and *post hoc* Sidak’s multiple comparisons test. Significance for AGS incidence was determined using Fisher’s exact test. AGS severity score distributions were tested for normality and found to be non-normal by Shapiro–Wilk test. These score distributions were then statistically compared using a Mann–Whitney *U* test for analysis of ordinal datasets with non-normal distributions. Significant differences in latency to first seizure were determined using unpaired two-tailed Student’s *t* test. Results of all statistical analyses are reported in detail in the statistical table (Table 1) and figure legends.

**Table 1.** Statistics table

Figure	Data structure	Statistical test	Sample size	Statistical data
Figure 1*B*, metabolic labelling of protein synthesis with 50 μM lovastatin/vehicle				
	Normally distributed	Two-way RM ANOVA	*N* = 12per group	Genotype: *p* = 0.0106
WT veh vs *Fmr1* KO veh	Normally distributed	Sidak’s *post hoc*	*N* = 12per group	CI: –0.2916 to –0.06786, *p* = 0.0032
WT 50 μM lovastatin vs *Fmr1* KO 50 μM lovastatin	Normally distributed	Sidak’s *post hoc*	*N* = 12per group	CI: –0.1716 to 0.05214, *p* = 0.3516
*Fmr1* KO vehicle vs *Fmr1* KO 50 μM lovastatin	Normally distributed	Sidak’s *post hoc*	*N* = 12per group	CI: 0.007476 to 0.2312, *p* = 0.0368
Figure 1*C*, metabolic labelling of protein synthesis with 1–5 μM simvastatin/vehicle				
	Normally distributed	Two-way RM ANOVA	*N* = 10per group	Treatment:*p* < 0.0001, genotype: *p* = 0.0294
WT veh vs KO veh	Normally distributed	Sidak’s *post hoc*	*N* = 10per group	CI: –0.3188 to 0.09835, *p* = 0.3451
WT veh vs KO veh	Normally distributed	Paired *t* test	*N* = 10per group	CI: 0.008558 to 0.2119, *p* = 0.0366
WT veh vs WT 5 μM simvastatin	Normally distributed	Sidak’s *post hoc*	*N* = 10per group	CI: –0.7435 to –0.3263, *p* = 0.0001
*Fmr1* KO veh vs *Fmr1* KO 5 μM simvastatin	Normally distributed	Sidak’s *post hoc*	*N* = 10per group	CI: –0.8045 to –0.3873, *p* < 0.0001
Figure 1*D*, metabolic labelling of protein synthesis with 0.1–0.5 μM simvastatin/vehicle				
	Normally distributed	Two-way RM ANOVA	*N* = 9per group	Treatment: *p* < 0.0001, genotype: *p* = 0.0068
WT veh vs *Fmr1* KO veh	Normally distributed	Sidak’s *post hoc*	*N* = 9per group	CI: –0.2483 to –0.06400, *p* = 0.0005
WT veh vs WT 0.3 μM simvastatin	Normally distributed	Sidak’s *post hoc*	*N* = 9per group	CI: –0.2760 to –0.07980, *p* = 0.0002
WT veh vs WT 0.5 μM simvastatin	Normally distributed	Sidak’s *post hoc*	*N* = 9per group	CI: –0.3394 to –0.1432,*p* < 0.0001
*Fmr1* KO veh vs *Fmr1* KO 0.3 μM simvastatin	Normally distributed	Sidak’s *post hoc*	*N* = 9per group	CI: –0.2334 to –0.03724,*p* = 0.0035
*Fmr1* KO veh vs *Fmr1* KO 0.5 μM simvastatin	Normally distributed	Sidak’s *post hoc*	*N* = 9per group	CI: –0.3121 to –0.1159,*p* < 0.0001
WT 0.1 μM simvastatin vs *Fmr1* KO 0.1 μM simvastatin	Normally distributed	Sidak’s *post hoc*	*N* = 9per group	CI: –0.1874 to –0.003152,*p* = 0.0406
WT 0.3 μM simvastatin vs *Fmr1* KO 0.3 μM simvastatin	Normally distributed	Sidak’s *post hoc*	*N* = 9per group	CI: –0.2057 to –0.02143,*p* = 0.0115
WT 0.5 μM simvastatin vs *Fmr1* KO 0.5 μM simvastatin	Normally distributed	Sidak’s *post hoc*	*N* = 9per group	CI: –0.2210 to –0.03669,*p* = 0.0038
Figure 2*B*, phospho/total ERK1/2 with 50 μM lovastatin/vehicle				
	Normally distributed	Two-way RM ANOVA	*N* = 19per group	Genotype: *p* = 0.0146
(Continued)				
WT veh vs *Fmr1* KO veh	Normally distributed	Sidak’s *post hoc*	*N* = 19per group	CI: –0.02577 to 0.1893,*p* = 0.1539
*Fmr1* KO veh vs *Fmr1* KO lovastatin	Normally distributed	Sidak’s *post hoc*	*N* = 19per group	CI: 0.04797 to 0.2630,*p* = 0.0048
Figure 2*C*, phospho/total ERK1/2 with 0.1–0.5 μM simvastatin/vehicle				
	Normally distributed	Two-way RM ANOVA	*N* = 11per group	Genotype: *p* = 0.7010, treatment: *p* = 0.8761
Figure 2*D*, phospho/total p70S6K with 0.1–0.5 μM simvastatin/vehicle				
	Normally distributed	Two-way RM ANOVA	*N* = 10per group	Genotype: *p* = 0.2860, treatment: *p* = 0.6206
Figure 3*B*, AGS incidence with 3 mg/kg simvastatin				
WT veh vs *Fmr1* KO veh	Non-normal distribution	Two-tailed Fisher’s exact test	*N* = 12per group	CI: 0.002672 to 0.3437, *p* = 0.0028
WT simvastatin vs *Fmr1* KO simvastatin	Non-normal distribution	Two-tailed Fisher’s exact test	*N* = 12per group	CI: 0.002918 to 0.3808, *p* = 0.0028
*Fmr1* KO veh vs *Fmr1* KO simvastatin	Non-normal distribution	Two-tailed Fisher’s exact test	*N* = 12per group	CI: 0.1915 to 5.221, *p* > 0.9999
Figure 3*C*, AGS severity distribution scores with 3 mg/kg simvastatin				
WT veh vs *Fmr1* KO veh	Non-normal distribution	Mann–Whitney test	*N* = 12per group	CI: 0.000 to 2.000, *p* = 0.0028
*Fmr1* KO veh vs *Fmr1* KO simvastatin	Non-normal distribution	Mann–Whitney test	*N* = 12per group	CI: –1.000 to 1.000, *p* = 0.9510
Figure 3*D*, AGS latency with 3 mg/kg simvastatin				
*Fmr1* KO veh vs *Fmr1* KO simvastatin	Normally distributed	Unpaired two-tailed *t* test	*N* = 12per group	CI: –11.56 to 43.11,*p* = 0.2388
Figure 3*E*, AGS incidence with 50 mg/kg simvastatin				
WT veh vs *Fmr1* KO veh	Non-normal distribution	Two-tailed Fisher’s exact test	KO veh: *n* = 14WT veh: *n* = 12	CI: 0.004960 to 0.5143, *p* = 0.0053
WT simvastatin vs *Fmr1* KO simvastatin	Non-normal distribution	Two-tailed Fisher’s exact test	KO simva: *n* = 11WT simva:*n* = 13	CI: 0.006556 to 0.7356, *p* = 0.0233
*Fmr1* KO veh vs *Fmr1* KO simvastatin	Non-normal distribution	Two-tailed Fisher’s exact test	KO veh: *n* = 14 KO simva: *n* = 11	CI: 0.2988 to 7.531, *p* = 0.6968
Figure 3*F*, AGS severity scores with 50 mg/kg simvastatin				
WT veh vs *Fmr1* KO veh	Non-normal distribution	Mann–Whitney test	KO veh: *n* = 14WT veh: *n* = 12	CI: 0.000 to 3.000, *p* = 0.0036
*Fmr1* KO veh vs *Fmr1* KO simvastatin	Non-normal distribution	Mann–Whitney test	KO veh: *n* = 14 KO simva: *n* = 11	CI: –3.000 to 0.000, *p* = 0.2254
Figure 3*G*, AGS latency with 50 mg/kg simvastatin				
*Fmr1* KO veh vs *Fmr1* KO simvastatin	Normally distributed	Unpaired two-tailed *t* test	KO veh: *n* = 14 KO simva: *n* = 11	CI: –11.41 to 8.739,*p* = 0.7794
Figure 3*H*, AGS incidence with 100 mg/kg lovastatin				
WT veh vs *Fmr1* KO veh	Non-normal distribution	Two-tailed Fisher’s exact test	KO veh: *n* = 16WT veh: *n* = 15	CI: 0.01126 to 0.4341, *p* = 0.0032
WT lovastatin vs *Fmr1* KO lovastatin	Non-normal distribution	Two-tailed Fisher’s exact test	KO lova: *n* = 14WT lova:*n* = 17	CI: 0.06948 to 3.440, *p* = 0.6358
(Continued)				
*Fmr1* KO veh vs *Fmr1* KO lovastatin	Non-normal distribution	Two-tailed Fisher’s exact test	KO veh: *n* = 16 KO lova: *n* = 14	CI: 1.538 to 42.32, *p* = 0.0136
Figure 3*I*, AGS severity distribution scores with 100 mg/kg lovastatin				
WT veh vs *Fmr1* KO veh	Non-normal distribution	Mann–Whitney test	KO veh: *n* = 16*n* = WT veh: *n* = 15	CI: 0.000 to 3.000, *p* = 0.0064
*Fmr1* KO veh vs *Fmr1* KO lovastatin	Non-normal distribution	Mann–Whitney test	KO veh: *n* = 16 KO lova: *n* = 14	CI: –3.000 to 0.000, *p* = 0.0204
Figure 3*J*, AGS latency with 100 mg/kg lovastatin				
*Fmr1* KO veh vs *Fmr1* KO simvastatin	Normally distributed	Unpaired two-tailed *t* test	KO veh: *n* = 16 KO lova: *n* = 14	CI: 3.595 to 31.07,*p* = 0.0176

## Results

### Lovastatin, but not simvastatin, normalizes excessive protein synthesis in the *Fmr1^-/y^* hippocampus

Previous work shows that lovastatin normalizes excessive protein synthesis in the *Fmr1^-/y^* hippocampus through reduction of Ras-ERK1/2 activation, which corrects epileptogenic phenotypes (Osterweil et al., 2013). To examine whether the same effect is seen with simvastatin, we used a metabolic labeling assay in hippocampal slices designed to assess protein synthesis in an intact preparation under physiologic conditions. Hippocampal slices were prepared from juvenile WT and *Fmr1^-/y^* littermates, blind to genotype, and allowed to recover in oxygenating ACSF. Following this, slices were preincubated with Actinomycin D to block transcription, and new protein synthesis was labeled through incorporation of ^35^S-labeled methionine/cysteine mix (Fig. 1*A*).

Previous experiments tested a range of 10–50 µM lovastatin and showed that 50 µM was effective in normalizing protein synthesis in the *Fmr1^-/y^* hippocampus (Osterweil et al., 2013). To ensure that we could recapitulate these results, we measured protein synthesis in WT and *Fmr1^-/y^* slices ±50 µM lovastatin (Fig. 1*B*). As expected, our experiments revealed a significant correction of excessive protein synthesis with lovastatin in the *Fmr1^-/y^* mouse (WT veh = 100 ± 1.48%, WT lova = 100.06 ± 4.87%, KO veh = 117.97 ± 4.27%, KO lova = 106.04 ± 4.93%; WT vs KO veh *p* = 0.0032, KO veh vs lova *p* = 0.0368; *n* = 12). Next, we tested the efficacy of simvastatin using the same assay system. As simvastatin is twice as potent as lovastatin with respect to reducing plasma LDL cholesterol levels in patients, we tested a lower dose range of simvastatin in our metabolic labeling assay (Tsuji et al., 1993; Jones et al., 1998; Schaefer et al., 2004; Neuvonen et al., 2008). This concentration is consistent with previous studies of simvastatin in cultured neurons (Lim et al., 2006; Johnson-Anuna et al., 2007; Mans et al., 2010). Interestingly, we find that simvastatin treatment not only fails to reduce protein synthesis in the *Fmr1^-/y^* hippocampus, it causes a significant increase in both WT and *Fmr1^-/y^* slices at 5 µM (WT vehicle = 100 ± 2.70%, WT 5 μM = 153.5 ± 6.32%, KO vehicle = 111 ± 4.27%, KO 5 μM = 170.60 ± 9.43%; WT veh vs 5 μM *p* = 0.0001, KO veh vs 5 μM *p* < 0.0001; *n* = 10; Fig. 1*C*).

This puzzling increase in protein synthesis led us to wonder whether a reduced concentration of simvastatin might be more appropriate. To test this, we exposed slices to vehicle or simvastatin at concentrations of 0.1–0.5 µM. Surprisingly, we find that even at these lower concentrations simvastatin causes a dose-dependent increase in protein synthesis, worsening the *Fmr1^-/y^* phenotype (WT veh = 100 ± 2.21%, WT 0.1 μM = 106.99 ± 3.51%, WT 0.3 μM = 117.79 ± 4.08%, WT 0.5 μM = 124.13 ± 4.23%, KO veh = 115.61 ± 3.48%, KO 0.1 μM = 116.52 ± 2.21%, KO 0.3 μM = 129.15 ± 3.99%, KO 0.5 μM = 137.01 ± 3.08%; WT veh vs 0.3 μM *p* = 0.0002, WT veh vs 0.5 μM *p* < 0.0001, KO veh vs 0.3 μM *p* = 0.0035, KO veh vs 0.5 μM *p* < 0.0001; *n* = 9; Fig. 1*D*). These results show that unlike lovastatin, simvastatin does not correct excessive protein synthesis in the *Fmr1^-/y^* hippocampus.

### Lovastatin, but not simvastatin, reduces ERK1/2 activation

Our metabolic labeling experiments show that 50 µM lovastatin reduces protein synthesis in the *Fmr1^-/y^* hippocampus by 15–20% (Fig. 1*B*). Conversely, 0.5 µM simvastatin causes a 15–20% increase in protein synthesis in the *Fmr1^-/y^* hippocampus (Fig. 1*D*). Given the opposite effect of lovastatin and simvastatin on protein synthesis, we wondered whether these compounds acted differently on the ERK1/2 and mTOR translation control signaling pathways (Fig. 2*A*). To confirm the same lovastatin treatment that reduces excess protein synthesis in the *Fmr1^-/y^* also reduces ERK1/2 activation, we incubated slices in vehicle or 50 µM lovastatin and performed quantitative immunoblotting for p-ERK1/2 (Fig. 2*B*; Extended Data Fig. 2-1). Our results confirm that 50 µM lovastatin significantly reduces p-ERK1/2 in *Fmr1^-/y^* slices as previously reported (WT veh = 100 ± 4.32%, WT lova = 99.28 ± 4.42%, KO veh = 91.83 ± 4.74%, KO lova = 76.28 ± 3.76%; KO veh vs lova *p* = 0.0048; *n* = 19).

Next, to test whether simvastatin had a differential impact on ERK1/2 signaling at the same concentration that causes a 15–20% increase in protein synthesis, we repeated our immunoblotting analysis on slices exposed to vehicle or 0.1–0.5 µM simvastatin. In contrast to lovastatin, our results show that simvastatin has no significant impact on p-ERK1/2 in either WT or *Fmr1^-/y^* slices at any dose tested (WT veh = 100 ± 4.51%, WT 0.1 μM = 102.87 ± 3.42%, WT 0.3 μM = 108.45 ± 4.10%, WT 0.5 μM = 101.01% ± 2.09%, KO veh = 105.63 ± 4.97%, KO 0.1 μM = 98.94 ± 4.46%, KO 0.3 μM = 94.71 ± 4.53%, KO 0.5 μM = 106.93 ± 3.65%; *n* = 11; Fig. 2*C*; Extended Data Fig. 2-1). This suggests that simvastatin neither activates nor inhibits the ERK1/2 pathway under conditions where it increases protein synthesis.

Although our previous study with lovastatin showed no effect of lovastatin on mTOR activation as assessed by phosphorylation of p70S6K, we wondered whether simvastatin had an observable impact on this pathway. To investigate, we immunoblotted for p-p70S6K in WT and *Fmr1^-/y^* slices treated with 0.1–0.5 µM simvastatin. Our results show that p70S6K activation is unchanged in slices treated with 0.1–0.5 µM simvastatin (WT veh = 100 ± 11.14%, WT 0.1 μM = 112.94 ± 10.25%, WT 0.3 μM = 110.66 ± 9.47%, WT 0.5 μM = 98.89 ± 4.72%, KO veh = 92.87 ± 4.49%, KO 0.1 μM = 85.37% ± 11.82%, KO 0.3 μM = 101.71% ± 10.37%, KO 0.5 μM = 92.53% ± 10.64%; *n* = 10; Fig. 2*D*; Extended Data Fig. 2-1). Together, these experiments show that unlike lovastatin, simvastatin does not affect the activation of ERK1/2, nor does it alter the mTORC1-p70S6K pathway.

### Lovastatin, but not simvastatin, corrects the AGS phenotype in the *Fmr1^-/y^* mouse

Our work *in vitro* shows that simvastatin does not correct the ERK1/2-stimulated excess in protein synthesis in the *Fmr1^-/y^* hippocampus, suggesting that it may not have the same efficacy as lovastatin in ameliorating pathologic phenotypes. To directly test this, we performed a side-by-side analysis of the effect of lovastatin versus simvastatin on the incidence of AGS in the *Fmr1^-/y^* mouse. Although the AGS phenotype is seen in *Fmr1^-/y^* mice bred on multiple mouse background strains, a more robust phenotype is observed in mice bred on the FVB strain or a C57Bl6/J x FVB hybrid strain (Yan et al., 2004, 2005). Therefore, we used *Fmr1^-/y^* and littermate WT mice bred on a C57Bl6/J x FVB hybrid strain for our AGS study. Importantly, lovastatin corrects the AGS phenotype in *Fmr1^-/y^* bred on both C57BL/6J and FVB strains, suggesting the rescue is not dictated by background genetics (Osterweil et al., 2013).

To test whether simvastatin could similarly correct the AGS phenotype, we injected *Fmr1^-/y^* and littermate WT mice with 3 mg/kg simvastatin as described in Materials and Methods. We used the lactone prodrug version of simvastatin administered to human patients, which is hydrolyzed into the active hydroxy acid compound by the liver (Schachter, 2005). The initial dose of simvastatin was chosen based on previous work showing 1 mg/kg simvastatin reduces epileptogenic activity and neurotoxicity in a kainic acid (KA) rat model of epilepsy (Xie et al., 2011). Additionally, according to a conversion factor of 0.081 for mouse to human dosing recommended by the Food and Drug Administration (FDA), 3 mg/kg simvastatin in mouse would be equivalent to the 20 mg dose used in humans (Nair and Jacob, 2016).

**Figure 3.**
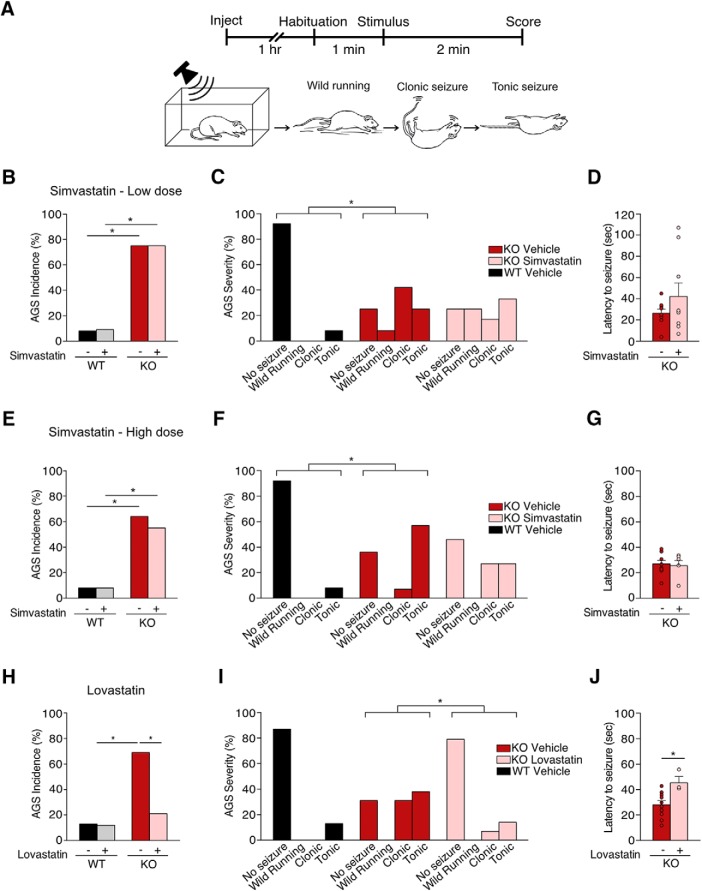
Simvastatin does not correct AGS in the *Fmr1^-/y^* mouse. *Fmr1^-/y^* and littermate WT mice were injected intraperitoneally with vehicle, simvastatin, or lovastatin and tested for AGS. ***A***, Schematic shows the experimental timeline and scoring system for AGS testing. ***B***, Injection of 3 mg/kg simvastatin does not reduce the incidence of AGS in *Fmr1^-/y^* mice (Fisher’s exact test WT vs KO veh *p = 0.0028, WT vs KO simva *p = 0.0028, KO veh vs simva *p* > 0.999). ***C***, Comparison of AGS scores also shows no reduction of seizure severity with 3 mg/kg simvastatin (Mann–Whitney WT vs KO veh *p = 0.0028, KO veh vs KO simva *p* = 0.9510). ***D***, 3 mg/kg simvastatin does not increase latency to first seizure in *Fmr1^-/y^* mice (unpaired *t* test *p* = 0.239). ***E***, 50 mg/kg active simvastatin does not reduce AGS incidence in *Fmr1^-/y^* mice (Fisher’s exact test WT vs KO veh *p = 0.0053, WT vs KO simva *p = 0.0233, KO veh vs simva *p* = 0.6968). ***F***, AGS severity scores are not significantly reduced with 50 mg/kg simvastatin (Mann–Whitney WT vs KO veh *p = 0.0036, KO veh vs KO simva *p* = 0.2254). ***G***, Latency to first seizure is not significantly different between vehicle and 50 mg/kg simvastatin-treated *Fmr1^-/y^* mice (unpaired *t* test *p* = 0.779). ***H***, Injection of 100 mg/kg lovastatin significantly reduces the incidence of AGS in *Fmr1^-/y^* mice (Fisher’s exact test WT vs KO veh *p = 0.0032, WT vs KO lova *p* = 0.6358, KO veh vs lova *p = 0.0136). ***I***, Lovastatin reduces severity scores of AGS in *Fmr1^-/y^* mice versus vehicle (Mann–Whitney WT vs KO veh *p = 0.0064, KO veh vs KO lova *p = 0.0204). ***J***, Lovastatin treatment significantly increases the latency to first seizure compared to vehicle-treated *Fmr1^-/y^* mice (unpaired *t* test KO veh vs lova *p = 0.0176). Error bars = SEM. *Figure Contributions*: Melania Muscas performed the experiments and analyzed the data.

Animals were injected with vehicle or simvastatin with the experimenter blind to genotype and treatment, and then left in a quiet environment for 1 h before AGS testing. A 1-h incubation time was chosen based on previous experiments using lovastatin, and on previous pharmacokinetic studies in mice and rats showing that simvastatin peaks in blood at 30 min to 1 h after administration (van de Steeg et al., 2013; Higgins et al., 2014; Xu et al., 2014), and peaks in brain 1 h after administration (Johnson-Anuna et al., 2005). To induce AGS, animals were transferred to a test chamber and exposed to a 2-min digitized sampling of a personal alarm passed through 50-W speakers at a level of >130 dB. Seizures were recorded at increasing levels of severity as: 1, wild running (uncontrolled and undirected running); 2, clonic seizure (loss of balance with violent spasms on all limbs); and 3, tonic seizure (loss of balance with postural rigidity in limbs and tail; Fig. 3*A*). Latency between the onset of the AGS stimulus and seizure was also used as a metric of seizure severity and measured as the number of seconds between the start of the alarm to the first appearance of wild running.

Our results show that vehicle-treated *Fmr1^-/y^* mice exhibit a significantly higher incidence of AGS versus WT littermates (WT veh 8%, KO veh 75%, *p* = 0.0028) and a significant increase in seizure severity (WT vs KO veh *p* = 0.0028). However, in contrast to lovastatin, 3 mg/kg simvastatin injection had no significant effect on the incidence of AGS in *Fmr1^-/y^* mice (WT veh 8%, WT simva 9%, KO veh 75%, KO simva 75%; WT vs KO simva *p* = 0.0028, KO veh vs simva *p* = 1.000; Fig. 3*B*). Comparison of AGS scores showed 3 mg/kg simvastatin was similarly ineffective in reducing seizure severity (KO veh: wild running 1/12, clonic 5/12, tonic 3/12; KO simva: wild running 3/12, clonic 2/12, tonic 4/12; KO veh vs simva *p* = 0.951; Fig. 3*C*). Measurements of the latency to first seizure also reveal no significant effect of simvastatin treatment (KO veh = 26.33 ± 3.80 s, KO simva = 42.11 ± 12.32 s, *p* = 0.239; Fig. 3*D*). These results suggest simvastatin is not effective in correcting AGS in *Fmr1^-/y^* mice.

Although 3 mg/kg is consistent with a simvastatin dose used in previous studies of KA-induced seizure, higher doses of up to 50 mg/kg have also been investigated with respect to neurologic phenotypes in rodents (Ramirez et al., 2011; Ling and Tejada-Simon, 2016). Indeed, intraperitoneal injection of 50 mg/kg active simvastatin 24 h and 30 min before seizure induction protects against KA-induced seizures in mice (Ramirez et al., 2011), and increases learning in a mouse model of Alzheimer’s disease (Li et al., 2006). To ensure that simvastatin is not effective in correcting the AGS phenotype in *Fmr1^-/y^* mice, we repeated our experiments using a high dose of 50 mg/kg. To remove the potential confound of prodrug metabolism, we injected active simvastatin hydroxy acid rather than inactive lactone. In a comparison group, we tested an equipotent 100 mg/kg dose of active lovastatin hydroxy acid that was previously shown to correct AGS in adult *Fmr1^-/y^* FVB mice (Osterweil et al., 2013). Separate groups of *Fmr1^-/y^* and WT littermates were injected with 50 mg/kg simvastatin or 100 mg/kg lovastatin (with corresponding vehicle) and AGS testing performed as previously.

Our results show that even at a higher dose, simvastatin does not reduce AGS incidence in *Fmr1^-/y^* mice (WT veh 8%, WT simva 8%, KO veh 64%, KO simva 55%; WT vs KO veh *p* = 0.0053, WT vs KO simva *p* = 0.0233, KO veh vs simva *p* = 0.6968; Fig. 3*E*). AGS severity is similarly not reduced in simvastatin-treated *Fmr1^-/y^* mice as assessed by seizure score (KO veh: wild running 0/14, clonic 1/14, tonic 8/14; KO simva: wild running 0/11, clonic 3/11, tonic 3/11; WT vs KO veh *p = 0.0036, KO veh vs KO simva *p* = 0.2254; Fig. 3*F*) or latency to seizure onset (KO veh = 27 ± 2.95 s, KO simva = 25.67 ± 3.61 s, *p* = 0.779; Fig. 3*G*). In contrast, *Fmr1^-/y^* mice injected with 100 mg/kg lovastatin showed a significant reduction in AGS versus vehicle-treated mice (WT veh 13%, WT lova 12%, KO veh 69%, KO lova 21%; WT vs KO veh *p* = 0.0032, KO veh vs lova *p* = 0.0136, WT veh vs KO lova *p* = 0.6513; Fig. 3*H*). Additionally, AGS scoring reveals a decrease in the severity of seizures in lovastatin-treated *Fmr1^-/y^* mice (KO veh: wild running 0/16, clonic 5/16, tonic 6/16; KO lova: wild running 0/14, clonic 1/14, tonic 2/14; WT vs KO veh *p = 0.0064, KO veh vs KO lova *p = 0.0204; Fig. 3*I*), and an increase in the latency to the first seizure (KO veh = 28 ± 3 s, KO lova = 45.33 ± 4.84 s, KO veh vs lova *p = 0.0176; Fig. 3*J*). Together, these results show that lovastatin reduces the incidence and severity of AGS in the *Fmr1^-/y^*, whereas simvastatin has no effect.

## Discussion

The promising results using lovastatin in FX have led to the suggestion that simvastatin may be similarly effective. In this study, we investigated two core phenotypes in the *Fmr1^-/y^* mouse model to test the prediction that simvastatin can be used in place of lovastatin. Our results show that simvastatin not only fails to correct excessive protein synthesis in the *Fmr1^-/y^* hippocampus, it worsens this phenotype (Fig. 1). We do not see a reduction of ERK1/2 activation at the concentrations of simvastatin tested (Fig. 2). Moreover, simvastatin does not reduce the incidence or severity of AGS in the *Fmr1^-/y^* mouse even at a high dose of 50 mg/kg (Fig. 3). These results suggest that simvastatin should not be assumed to be an effective replacement for lovastatin with respect to correction of *Fmr1^-/y^* pathology.

Although we propose the beneficial effect of lovastatin stems from the inhibition of ERK1/2-driven protein synthesis, it is important to note that statins are capable of affecting several biochemical pathways. Beyond the canonical impact on cholesterol biosynthesis, statins also decrease isoprenoid intermediates including farnesyl and geranylgeranyl pyrophosphates that regulate membrane association for many proteins including the small GTPases Ras, Rho, and Rac (Schafer et al., 1989; Liao and Laufs, 2005; Nürenberg and Volmer, 2012; Ling and Tejada-Simon, 2016). The increase in protein synthesis seen with simvastatin could be linked to altered posttranslational modification of these or other proteins. Indeed, although we see no change in mTORC1-p70S6K signaling, other studies have shown an activation of the PI3 kinase pathway that could be contributing to this effect (Mans et al., 2010). However, our comparison of lovastatin and simvastatin shows that there is a clear difference in the correction of pathology in the *Fmr1^-/y^* model, suggesting that the impact on ERK1/2 is an important factor in terms of pharmacological treatment for FX.

There are many reasons why statins would be an attractive option for treating neurodevelopmental disorders such as FX. They are prescribed worldwide for the treatment of hypercholesterolemia and coronary heart disease (Istvan, 2003), and safely used for long-term treatment in children and adults (Ling and Tejada-Simon, 2016). However, our study suggests that care should be taken when considering which statin should be trialed for the treatment of FX and other disorders of excess Ras. Although the effect of different statins on cholesterol synthesis has been well documented, the differential impact on Ras-ERK1/2 signaling is not well established. We show here that, contrary to lovastatin, simvastatin fails to inhibit the Ras-ERK1/2 pathway in the *Fmr1^-/y^* hippocampus, exacerbates the already elevated protein synthesis phenotype, and does not correct the AGS phenotype. These results are significant for considering future studies with lovastatin or simvastatin in FX or other disorders of excess Ras. Indeed, clinical trials using simvastatin for the treatment of NF1 have shown little promise, while trials with lovastatin show an improvement in cognitive deficits (van der Vaart et al., 2013; Bearden et al., 2016; Payne et al., 2016). Although further studies testing a broader dose range of simvastatin on additional *Fmr1^-/y^* brain phenotypes will ultimately determine the feasibility of this strategy for FX, our study suggests caution should be used when assuming simvastatin is a suitable substitute for lovastatin.

## Synthesis

Reviewing Editor: Quentin Pittman, University of Calgary Decisions are customarily a result of the Reviewing Editor and the peer reviewers coming together and discussing their recommendations until a consensus is reached. When revisions are invited, a fact-based synthesis statement explaining their decision and outlining what is needed to prepare a revision will be listed below. The following reviewer(s) agreed to reveal their identity: Kimberly Huber.

In this manuscript authors describe the effects of two statins, lovastatin and simvastatin, in well-described phenotypes of the Fmr1 knockout mouse, a model for fragile X syndrome.

The study finds that simvastatin does not reproduce the effects observed for lovastatin. The effects of lovastatin in the fragile X syndrome mouse model were previously published, and they are mainly reproduced in the present manuscript. Comparatively, simvastatin exposure, under similar experimental conditions than lovastatin, does not produce the same effects, although both drugs are HMG-CoA reductase inhibitors.

The reviewers agree that the findings are important for translational medicine. However, there was some disappointment that the authors did not take advantage of their data to explore alternative mechanisms, since their new data with simvastatin could indicate that lovastatin effects are independent of HMG-CoA reductase inhibition. While eNeuro does not demand elucidation of a mechanism for publication, if the authors have any evidence with respect to this matter they are encouraged to include it in a revision.

Suggestions for revision:

1. Regarding the main text, this is well written but the results section of the manuscript at times serves as an extended section of the introduction, the methods and the discussion sections (first half of page 9 would better fit in the introduction, where mTORC1 pathway is not mentioned; page 12, lines 1-11 would better fit in the methods section; page 11 and other parts of the results section would better fit in the discussion section). The results section should concisely describe the experimental data, and the rest of information should be redistributed.

2. Regarding the experimental data, Figure 1C does not show the phenotype between WT and Fmr1 KO mice, revealing some inconsistencies with the results previously published and those results shown in Figure 1 (B and D). Similarly, in the present experiments the enhanced ERK phosphorylation in basal conditions (Fmr1KO vs WT, in zero concentration of the drugs), previously published by other authors describing the effect of lovastatin, is not observed (Figure 2, B and C), raising questions about the performance of the assay.

3. Other minor drawback is the representative image chosen in Figure 1D. In the representative image for 35S detection, the WT 0 condition and WT 0.5 condition do not seem to reflect the averaged differences observed in the bars plot when comparing the darkest gray column and the lightest gray column.

4. Please detail in the methods how the protein synthesis and _pERK values are normalized. Are all values normalized to WT veh or to vehicle within the same genotype?

5. From the representative western blots in Fig. 2, the appropriate inclusion of a line between the WT and the KO indicates that each lane is cut or separated from adjacent lanes, suggesting that the authors “picked” lanes from different gels or blots. The authors should show a single blot with all conditions or at least all conditions from one genotype. The authors should also include molecular weight indicators on the gels. This is because often the P-p70S6K antibodies also recognize P-p85 S6K and it is important to be sure that the authors are measuring P-p70 band.

## Author Response

Dear Quentin,

Thank you very much for your careful consideration of our study and for passing along feedback from the reviewers. In response to these comments we have made a number of changes to the manuscript and included additional data that we believe greatly improve the manuscript. All amendments to the manuscript are highlighted, and Figure 1 has been changed to include our new data. We have also created Extended Data Figure 2-1 to show the original blots used for Figure 2, as requested by the reviewers, and full statistical table has been provided. We have also added RRIDs and figure legend contributions. A point-by-point response is below.

We feel these changes have significantly benefitted the manuscript and hope that this improved version will be accepted for publication in eNeuro. We look forward to hearing from you soon.

Sincerely,

Emily K. Osterweil, Ph.D.

Centre for Discovery Brain Sciences, Patrick Wild Centre

University of Edinburgh, Hugh Robson Building

George Square, Edinburgh, EH8 9XD

Email: Emily.osterweil@ed.ac.uk

1. Regarding the main text, this is well written but the results section of the manuscript at times serves as an extended section of the introduction, the methods and the discussion sections (first half of page 9 would better fit in the introduction, where mTORC1 pathway is not mentioned; page 12, lines 1-11 would better fit in the methods section; page 11 and other parts of the results section would better fit in the discussion section). The results section should concisely describe the experimental data, and the rest of information should be redistributed.

We thank the reviewer for pointing this out. The manuscript has now been streamlined to make the Results more succinct and move background information to the Introduction. All changes are highlighted.

2.Regarding the experimental data, Figure 1C does not show the phenotype between WT and Fmr1 KO mice, revealing some inconsistencies with the results previously published and those results shown in Figure 1 (B and D). Similarly, in the present experiments the enhanced ERK phosphorylation in basal conditions (Fmr1KO vs WT, in zero concentration of the drugs), previously published by other authors describing the effect of lovastatin, is not observed (Figure 2, B and C), raising questions about the performance of the assay.

The reviewer is correct that a genotype difference in protein synthesis did not pull out of the dataset shown in Figure 1C. We feel this is due to insufficient sample size in that dataset and have performed additional experiments for vehicle vs 5 µM simvastatin and the genotype effect has become significant by ANOVA (see revised Figure 1C). A post-hoc paired t-test shows a significant difference between vehicle WT and KO (provided in the statistical table).

Regarding the difference in p-ERK1/2 in WT versus Fmr1-/y hippocampus, previous work has shown that this is in fact not different between genotypes (Osterweil et al., 2010; Osterweil et al., 2013). These studies show that the greater impact of ERK1/2 and mGlu5 inhibitors on protein synthesis in the Fmr1-/y model is not due to a hyperactivation of the singaling cascade, but is rather due to a hypersensitive response to the same amount of ERK1/2 activation. The results shown in Figure 2 are consistent with these previous studies.

3. Other minor drawback is the representative image chosen in Figure 1D. In the representative image for 35S detection, the WT 0 condition and WT 0.5 condition do not seem to reflect the averaged differences observed in the bars plot when comparing the darkest gray column and the lightest gray column.

We have now revised this figure to include images that emphasize the difference between vehicle and 0.5 µM simvastatin.

4. Please detail in the methods how the protein synthesis and _pERK values are normalized. Are all values normalized to WT veh or to vehicle within the same genotype?

Protein synthesis is calculated by liquid scintillation counting from TCA-precipitated samples, with CPM divided by total protein quantified by Protein Concentration assay. The phosphorimages shown in Figure 1 are for representative purposes only.

To compare phopho- to total for each target in the same lane, membranes developed for phospho (i.e., p-ERK1/2) were stripped and re-probed for total (i.e., ERK1/2). Phosphorylation of target proteins was calculated as a ratio of phospho- to total. To correct for blot-to-blot variance, each signal was normalized to the average signal of all lanes on the same blot. Our gels are run such that each contains yoked pairs of WT-KO and veh-drug, ensuring that the average is not skewed by any one condition. Values are shown as a percentage of average WT vehicle for graphical purposes.

These details have been added to the Materials and Methods section.

5. From the representative western blots in Fig. 2, the appropriate inclusion of a line between the WT and the KO indicates that each lane is cut or separated from adjacent lanes, suggesting that the authors “picked” lanes from different gels or blots. The authors should show a single blot with all conditions or at least all conditions from one genotype. The authors should also include molecular weight indicators on the gels. This is because often the P-p70S6K antibodies also recognize P-p85 S6K and it is important to be sure that the authors are measuring P-p70 band.

We apologize for the confusion. Our samples were taken from the same blots, however we load these samples blind to genotype and treatment and they were therefore rearranged for the figure. The original membranes are now presented in Figure 2-1 showing the original sample loading.

We are aware of the issue with the p-p70S6K antibody and we therefore adopt a membrane cutting strategy to avoid the p85S6K antigen. Membranes are cut at 75KDa and 50KDa and the portion of membrane above 75KDa is removed to eliminate the background p85S6K band. This strategy is now explained in the Materials and Methods and we have included images of the membranes in Figure 2-1.
