# Supplementary figures and images for: Lovastatin, not Simvastatin, Corrects Core Phenotypes in the Fragile X Mouse Model
Source: eNeuro. 2019 Jun 10;6(3):ENEURO.0097-19.2019. doi: 10.1523/ENEURO.0097-19.2019 (PMC6565377; doi:10.1523/ENEURO.0097-19.2019)

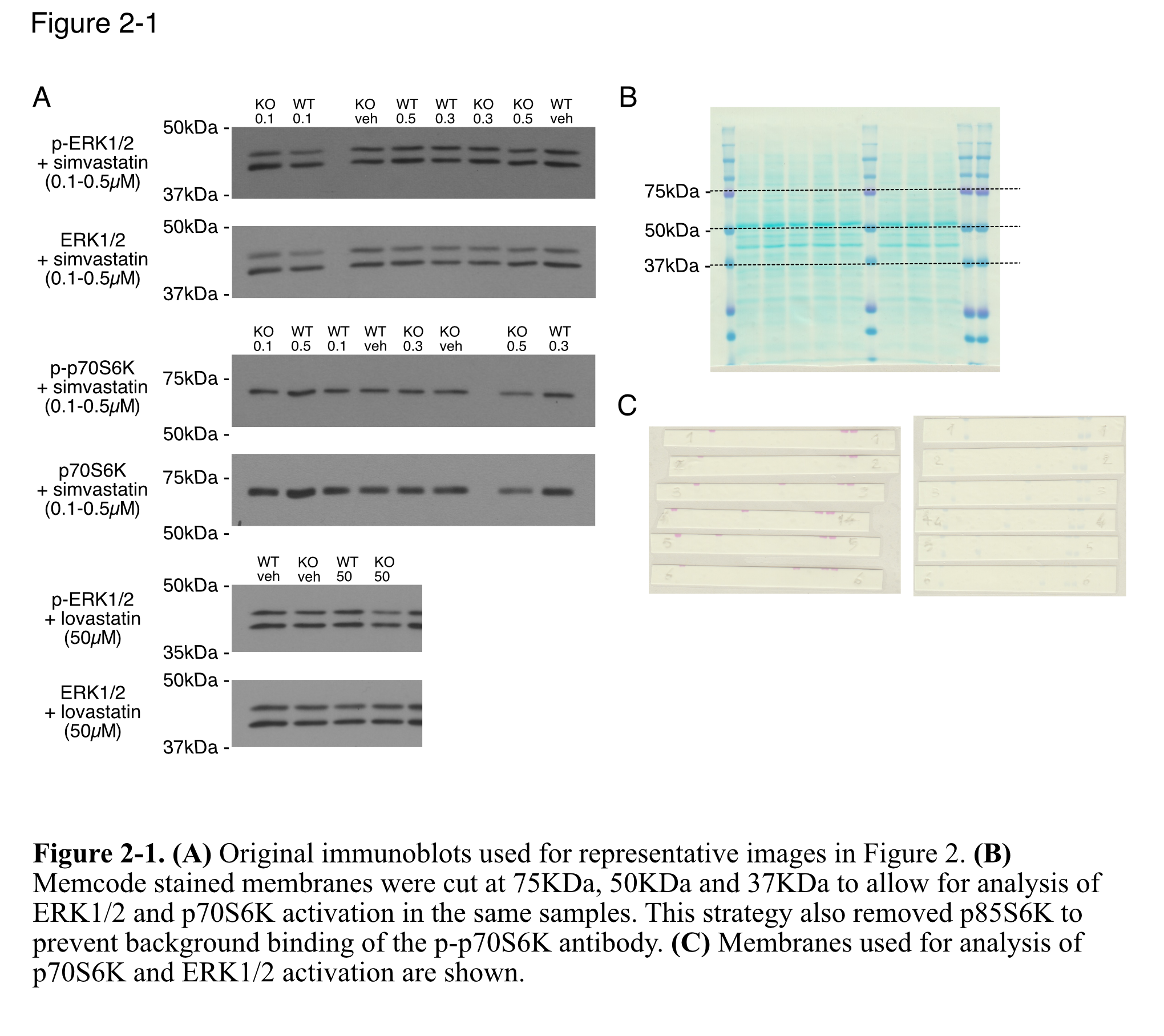

Supplement: Extended Data Figure 2-1 — A, Original immunoblots used for representative images in Figure 2. B, Memcode-stained membranes were cut at 75 , 50, and 37 kDa to allow for analysis of ERK1/2 and p70S6K activation in the same samples. This strategy also removed p85S6K to prevent background binding of the p-p70S6K antibody. C, Membranes used for analysis of p70S6K and ERK1/2 activation are shown. Figure Contributions: Melania Muscas and Susana R. Louros performed the experiments and analyzed the data. Download Figure 2-1, TIF file. [file sup_enu-eN-NWR-0097-19-s03.tif]
